# The soil bacterial and fungal diversity were determined by the stoichiometric ratios of litter inputs: evidence from a constructed wetland

**DOI:** 10.1038/s41598-019-50161-9

**Published:** 2019-09-25

**Authors:** Yunmei Ping, Xu Pan, Wei Li, Jinzhi Wang, Lijuan Cui

**Affiliations:** 1 Institute of Wetland Research, Chinese Academy of Forestry, Beijing, 100091 China; 2 Beijing Key Laboratory of Wetland Services and Restoration, Beijing, 100091 China; 3 Beijing Hanshiqiao National Wetland Ecosystem Research Station, Beijing, 101399 China

**Keywords:** Microbial ecology, Wetlands ecology

## Abstract

Plant litter is an important component in wetland ecosystems, and the role of plant litter decomposition is considered to be important for wetland ecosystem functions and services. However, the consequences of litter inputs have seldom been experimentally tested in real ecosystems such as constructed wetlands (CWs). The enriched nutrients in CWs might weaken the role of litter inputs on soil carbon and nitrogen cycling. Here, we conducted a two-month field experiment to examine the effects of litter inputs on the soils in CWs. Our results showed that litter inputs significantly affected soil microbial (bacterial and fungi) diversities and properties (soil total nitrogen and nitrogen isotopes), and litter species with higher stoichiometry ratios, i.e. C/N, C/P and N/P led to higher microbial diversities. However, litter species had no or weak effects on microbial activities (CO_2_ and CH_4_ flux) or on the relative abundance of microbial communities, indicating that other environmental factors in such a CW might have stronger effects on those factors than litter inputs. These results highlighted the importance of submerged plant litter in nutrient-rich wetland ecosystems and provide potential tools for managers to improve the ecosystem functions and/or services via altering microbial diversities.

## Introduction

Plant litter as the end of primary production entering into detritus food chains plays an important role in wetland ecosystems, and its decomposition can recycle carbon and multiple nutrients, alter environmental variables, and affect wetland ecosystem functions and services^1–5^. Litter decomposition in wetland ecosystems refers to the respiration and assimilation of plant litter by microbes and invertebrates^6^, and it can be divided into three interlinked processes, i.e. leaching, fragmentation and microbial decay^7,8^. Moreover, litter decomposition are commonly considered to be affected by the quality of litter, associated soil microbes and invertebrates and the corresponding environmental factors^7,9–13^, but in wetlands such as stream and other freshwater ecosystems, litter inputs and biotic or abiotic factors are proven to be paramount^14^. Given the large variation in plant functional traits among plant species, litter decomposition rate can vary significantly among species due to the various ‘afterlife’ effects of litter traits (i.e. C/N, lignin, base cations and other decomposition related traits)^15–17^, and this interspecific variation in decomposition rates might lead to uncertainties in the effects of wetland plant litter on the soil or water qualities^18,19^, and thereby other organism in wetland ecosystems^20–22^.

Previous studies have proved the significant and diverse effects of plant litter on soil physio-chemical properties and microbial communities^18,23–28^, and there were plenty of evidence either from a specific ecosystem type at local scale or from different biomes at the global scale^29,30^. It has been proved that soil ecological processes, including soil C, N cycling, flux of CO_2_, CH_4_ are closely related to litter decomposition^31,32^. There are multiple pathways that plant litter can affect the soils in wetland ecosystems: (1) litter inputs can have various effects on soil invertebrates or microbes via different physical and chemical traits; (2) litter inputs can also provide food, microhabitat or shelter for soil microbes or other soil fauna^33,34^; (3) litter inputs can have negative effects via releasing leachate from litter which contains detrimental organic carbon and/or other compounds^35–37^. All above mechanisms indicated that plant litter traits as litter qualities can strongly influence the chemical and physical composition of litter inputs, and thereby their decomposability^15^ and lead to substantial consequences to wetland ecosystems. However, among various litter traits, litter stoichiometry might form the most important constraints of carbon: nitrogen ratios on soil microbial communities^38,39^ and hence act as the key trait to predict the effects of litter inputs on soil properties and microbial communities in wetlands^40^.

Moreover, the main methodology to test those effects of plant litter was firstly to sample soils from the field, and then either directly quantify the soil properties and microbial community composition or activity^23,25^; or subsequently set up new controlled experiments in the lab with the litterbag method or soil-litter mixing method^18,26,27,41^. For the latter case, knowledge about effects of litter mixing on soil is derived mostly from ‘indoor’ experiments carried out in strictly controlled environments, or via quantifying the litter mass loss and nutrient release^42,43^. However, very few investigators have addressed such effects of litter mixing on the wetland soils in relatively dynamic and unstable environments, such as constructed wetlands (CWs) with irregular waste-water inputs^19^. In such kind of constructed wetlands, other environmental factors might weaken the effects of litter inputs on the soil and thereby other soil organism, such as bacteria and fungi.

Now in this study, we set up a soil-litter mixing experiment (two months) in an ongoing constructed wetland located in Hanshiqiao wetland, Beijing, China (latitude: 40°07′21.0″, longitude: 116°48′56.7″). Our hypothesis is that the effects of litter inputs in such a constructed wetland might not be as overwhelming as those proven in indoor laboratory experiments, in which the related environmental factors are strictly controlled and relatively stable. We explored the effects of litter traits and incubation time on the soil properties of a constructed wetland including soil CO_2_/CH_4_ fluxes, total carbon (TC), total nitrogen (TN), carbon and nitrogen isotope changes (^13^C, ^15^N), as well as microbial diversities. The purpose of our work was to fully understand the role of plant litter in constructed wetlands and develop potential tools for the maintenance, improvement and management of constructed wetlands using plant litter materials.

## Results

### Effects of litter inputs on soil properties

**Figure 1:**
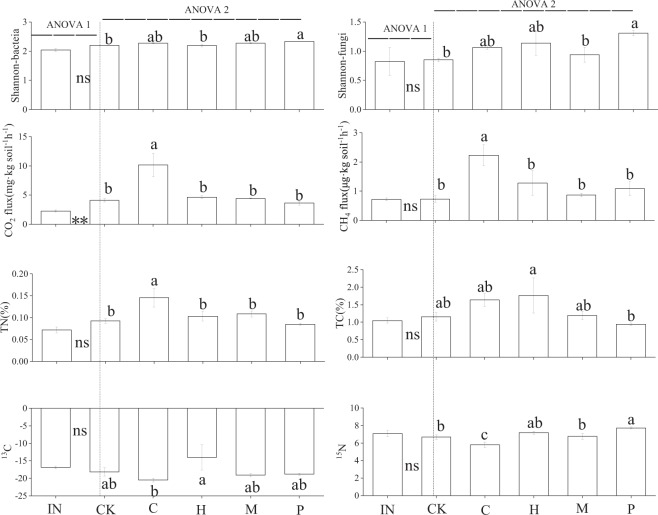
The soil microbial diversity, soil CO_2_, CH_4_ flux and characters before (IN: initial soil without litter) and after two-month incubation of different litter species (C: *Ceratophyllum demersum*; H: *Hydrilla verticillat*; M: *Myriophyllum verticillatum*; P: *Potamogeton crispus*). Values are means ± S.E. (n = 3). CK indicates the control treatment. The dotted line is used to separate two groups of ANOVA analyses: (1) ANOVA 1 showed the results between initial soil properties and the control treatment after two-month incubation (without litter mixing). **Indicated *P* < 0.01; ns indicated no significant differences between initial soil and CK. (2) ANOVA 2 showed the results among different litter species, including CK. Values by the same letter indicated no significant differences (*P* > 0.05).

There were significant differences between initial litter traits among four submerged plant species (Table 1). A significant difference was observed for CO_2_, but no significant difference in the other variables between initial soil and the control treatment (Fig. 1, ANOVA 1; Table S1). Moreover, litter of *Potamogeton crispus* significantly increased soil bacterial and fungi diversities, and ^15^N, but litter of *Ceratophyllum demersum* significantly increased soil TN, CO_2_ and CH_4_, but decreased ^15^N (Fig. 1, ANOVA 2; *P* < 0.05). However, litter species had no significant effects on either CO_2_ or CH_4_ (Fig. S1, Table S2).

**Table 1:** The initial traits (TN, TC, TP, C/N, C/P, N/P, ^13^C, ^15^N) of the four litter species (C: *Ceratophyllum demersum*; H: *Hydrilla verticillat*; M: *Myriophyllum verticillatum*; P: *Potamogeton crispus*) and initial soils (IN). Values are means ± S.E. (n = 3). Values within the same column followed by the same letter indicate no significant differences at *P* > 0.05.

Litter species and initial soil	TN%	TC%	TP%	C/N	C/P	N/P	^13^C	^15^N
*C*	2.37 ± 0.19b	24.36 ± 2.11bc	0.42 ± 0.43a	10.28 ± 0.15b	57.70 ± 0.11b	5.60 ± 0.01c	−27.16 ± 0.17d	3.97 ± 0.16d
*H*	2.51 ± 0.10b	22.76 ± 0.69c	0.49 ± 0.14a	9.08 ± 0.13b	46.20 ± 0.10b	5.00 ± 0.01c	−22.39 ± 0.39a	9.41 ± 0.07c
*M*	3.36 ± 0.07a	35.27 ± 0.91a	0.47 ± 0.21a	10.50 ± 0.04b	75.70 ± 0.22b	7.20 ± 0.02b	−25.83 ± 0.11c	10.20 ± 0.07b
*P*	1.81 ± 0.08c	29.87 ± 0.51ab	0.18 ± 0.15b	16.61 ± 0.94a	164.30 ± 1.47a	9.90 ± 0.05a	−24.46 ± 0.19b	19.19 ± 0.10a
IN	0.07 ± 0.00	1.05 ± 0.08	—	14.70 ± 0.39	—	—	−16.85 ± 0.40	7.08 ± 0.34

### Effects of litter inputs on soil microbial communities

**Figure 2:**
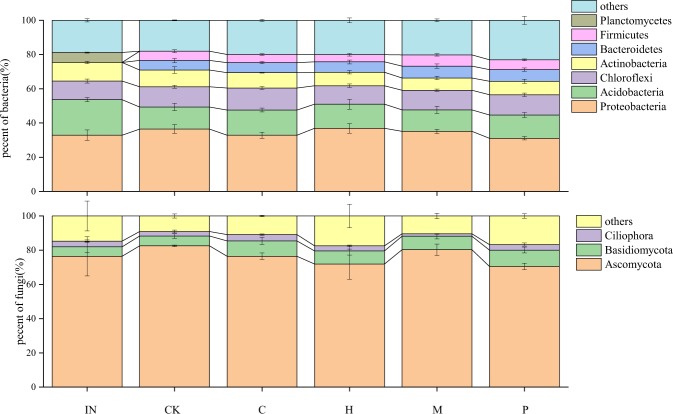
The relative abundance of bacteria and fungi in soil of different litter species before and after two-month incubation (IN: initial soil without litter mixing; CK: control treatment; C: *Ceratophyllum demersum*; H: *Hydrilla verticillat*; M: *Myriophyllum verticillatum*; P: *Potamogeton crispus*).

There were significant or marginally significant relationships between soil microbial diversity and litter stoichiometry ratios, i.e. C/N, C/P and N/P, but no significant relationships between soil microbial diversity and any other litter trait (Table 2). Moreover, after two-month incubation, one phylum of soil bacteria, i.e. *Planctomycetes*, significantly decreased, but another two phyla of soil bacteria, i.e. *Bacteroidetes* and *Firmicutes*, significantly increased (IN vs. CK: Fig. 2, *P* < 0.01). There were no significant differences in the soil fungi composition or the other phyla of soil bacteria before and after incubation (IN vs. CK: Fig. 2, *P* > 0.05). Overall, different species litter did not drive significant differences in the relative abundances of soil bacteria or fungi, except for some phyla (*Firmicutes* and *Ciliophora*) with lower relative abundance (<5%) (among four litter species and CK: Fig. 2, *P* > 0.05).

**Table 2:** Relationship between initial litter traits (C/N, C/P, N/P, TN, TC, TP, ^13^C and ^15^N) and the change value of soil microbial diversity (Shannon diversity index) before and after two-month incubation (the value = after − before). Values where *P* < 0.05 are in bold and *P* < 0.1 are in italic.

Shannon diversity index	Initial litter traits															
	C/N		C/P		N/P		TN%		TC%		TP%		^13^C		^15^N	
	*R*^2^	*P*	*R*^2^	*P*	*R*^2^	*P*	*R*^2^	*P*	*R*^2^	*P*	*R*^2^	*P*	*R*^2^	*P*	*R*^2^	*P*
bacteria	**0**.**90**	**0**.**01**	*0*.*64*	*0*.*07*	**0**.**91**	**0**.**01**	0.13	0.55	0.28	0.21	0.00	0.93	0.16	0.27	0.31	0.19
fungi	*0*.*67*	*0*.*06*	*0*.*60*	*0*.*08*	0.51	0.11	0.08	0.65	0.03	0.37	0.03	0.78	0.14	0.29	*0*.*59*	*0*.*08*

## Discussion

Litter inputs might affect the soil ecological processes, including soil C and N cycling via litter decomposition^31,32,44^. We indeed observed significant effects of litter inputs on soil properties (soil TN, ^13^C and ^15^N) and microbial diversities, and those effects to some extent depended on litter species identity (Fig. 1). These results indicated that in such a real wetland ecosystem mixing litter with soil still played an important role in regulating wetland soils including isotope signatures and affecting the soil microbial diversities. However, this was different from our original hypothesis, and in a way highlighted the importance of (even a small amount) litter inputs might have significant effects on wetland soils. Note that given the initial litter was cut into small pieces before mixing, the observed effects might be strengthened compared to real litter inputs from submerged plants, which is similar to previous laboratory studies^28,41,45^.

The different responses of wetland soils to litter mixing might result from the different reactions of micro-organisms to different litter traits and/or to different chemical fractions released during litter decomposition processes^46^. In our study, we observed the highest TN and the lowest ^15^N in the soil mixed with *C*. *demersum* litter after two-month incubation (Fig. 1), and this might be due to the lowest ^13^C and ^15^N in the initial litter of *C*. *demersum* (Table 1). Isotope signatures can represent the ratios of heavier element (^13^C and ^15^N) to lighter element (^12^C and ^14^N), to some extent determining the decomposition rates of plant species litter^47–49^. Moreover, we also observed the highest microbial diversities and the highest ^15^N in the soil mixed with *P*. *crispus* litter after two-month incubation, and this might also result from the stoichiometric or isotope ratios of initial *P*. *crispus* litter (Table 1). The imbalance among plant litter, microbial biomass and soil stoichiometry might explain this phenomenon^18,50^, but regrettably we did not have the data for microbial stoichiometry. Instead, we indeed found litter inputs might offset the imbalance between soil and mixed litter, and there was a significant positive correlation between litter stoichiometric ratios and microbial diversities (Table 2), indicating that litter traits related to stoichiometry ratios were still important predictors for the effects of litter inputs on soil C and N cycling^50^. However, we did not find significant effects of litter inputs on the relative abundances of microbes except for several minor groups (Fig. 2), and the relative abundance of microbes might largely be determined by the long-term waste water inputs rather than the short-term litter inputs.

**Figure 3:**
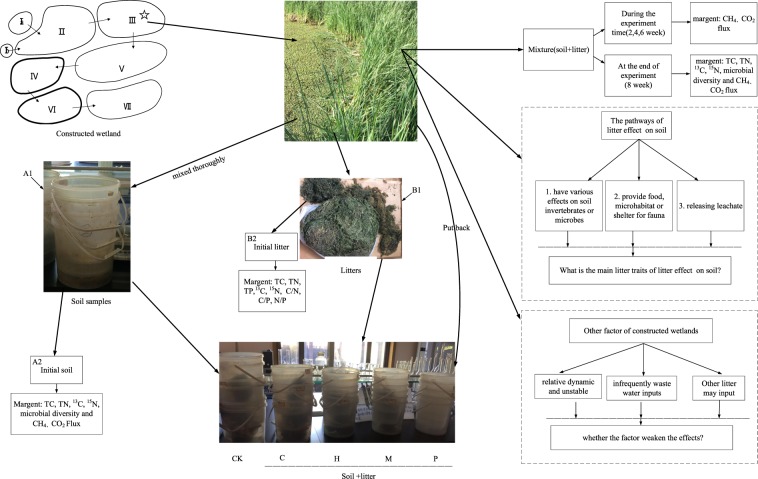
Conceptual framework and experimental arrangements for our study. Four litter species are: C: *Ceratophyllum demersum*; H: *Hydrilla verticillat*; M: *Myriophyllum verticillatum*; P: *Potamogeton crispus*; CK: control treatment. “ → ” in the constructed wetland is water flow direction.“⭐” is the study site.

Litter inputs might also affect the CO_2_ and CH_4_ flux via the interactions between microbes and litter sources^26,44,51^, and litter traits such as C/N ratios were expected to drive those differences^52,53^. However, our results showed no significant differences in CO_2_ and CH_4_ flux among plant species (Table S2), but only the *C*. *demersum* litter led to the highest CO_2_ and CH_4_ flux after two months (Fig. 1). There might be due to the weaker effects of litter mixing than other factors from the constructed wetlands (Fig. 3), such as temperature and the C/N ratios of the waste water inputs. The continuous inputs of wasted water with extra carbon and nutrients in our study site might be more overwhelming than the effects of such a small amount of litter inputs. Note that the quantity of plant litter and the time of incubation might also matter^27^, and we suggested that future studies should incorporate both litter quality and quantity effects and put them in a relatively longer incubation period.

## Conclusion

In conclusion, our results provided empirical evidence for the effects of submerged plant litter on the soil properties and microbial diversities in a constructed wetland. These findings might have multiple implications for the design, maintenance and management of constructed wetlands: (1) when designing a constructed wetland, it is better not only take the species identity into consideration, but also for the stoichiometry of different growth forms^19^; (2) for constructed wetlands, plant litter of submerged plants should not have been always considered as wastes and being directly refloated from the CWs. Instead, it is possible to shift the ‘unfavored’ submerged plant litter to ‘useful’ tools to improve the ecosystem functions and services of constructed wetlands; (3) plant litter might be a feasible and economic materials for improving the microbial diversities of CWs, and it is worth to comprehensively study the role of wetland plants in constructed wetlands, especially for the role of plant litter, and this might provide valuable suggestions for managers about the maintenance and management of constructed wetlands.

## Materials and Methods

### Study site

Our study site was located in the Hanshiqiao wetland, Beijing, China. There was a constructed wetland, which was used to purify the waste water from the pleasure boat area, restaurant and the public toilet in the Hanshiqiao wetland Park (Fig. 3). The CW consists of seven treatment sections, and we conducted a soil-litter mixing experiment in one section of a constructed wetland (CW), i.e. the section III as the study site (marked as ✩ in Fig. 3). The area of the section III is 582.30 m^2^. The water average depth is about 2.5 m. The dominant plant species in section III are *Iris wilsonii*, *Zizania latifolia*, *Typha orientalis* and *Sagittaria sagittifolia*, and there were irregular waste water inputs flowing into our study site through early April to late November every year.

### Experimental design

Plant litter of four submerged species (eg., *Ceratophyllum demersum*, *Myriophyllum verticillatum*, *Hydrilla verticillata*, *Potamogeton crispus*) were collected from Hanshiqiao wetland park in July 2017 (but not from the constructed wetland). All plant litter (Fig. 3, B1) was air-dried at room temperature for at least one month. The litter was subsequently cut into pieces (<5 mm) in order to increase the decomposition rate of litter and to maximize the effect of litter inputs on the soil in the CW. Initial soil samples (Fig. 3, A1) (upper 10 cm layer) were collected from five random locations in the CW by shovel, and then were thoroughly mixed. Any visible roots or contaminants were removed before the experiment. We prepared 15 plastic buckets (Fig. 3, the bottom diameter is around 22 cm and the height of the bucket is around 27.5 cm), and each bucket has only one litter species (litter species treatments) or no litter species (the control treatment). Within each plastic bucket, we put 400 g wetland soils and 4.0 g air-dried litter, and thoroughly mixed them in order to keep the dry-weight ratio of soil vs. litter consistently across treatments^18,27^. Finally, we randomly placed all the buckets at the bottom of our study site (submerged by water in the CW) and all the buckets were incubated in the same environment only with different litter input treatments. The distances between each bucket was around 30 cm. The whole experiment last from August 31, 2017 to November 2, 2017, which is also the main senesced period in the study region.

### Sampling and measurements

Before incubation, three soil samples (Fig. 3, A2) and five litter samples for each species (Fig. 3, B2) were selected for the initial soil properties and initial litter trait measurements. The initial soil and litter measurements (listed in Table 1) included total carbon (TC), total nitrogen (TN), soil total phosphorus (TP), and stable isotopes (^13^C and ^15^N). Total C and Total N content of were assessed using the VarioMAX CN element analyzer (Macro Elemental Analyzer System GmbH, Hanau, Germany). The TP concentration was analyzed by inductively coupled plasma emission spectroscopy (Perkin Elmer Optima 3000 ICP Spectrometer, Waltham, MA, USA) and the isotopes of C and N were subsequently analyzed using an isotope ratio mass spectrometer (Isoprime100; Isoprime Ltd, UK).

Initial soil microbe community, i.e. bacteria and fungi, was a measured using the second generation next throughput sequencing technology (MiSeq high throughput sequencing, 16Sr DNA sequences). Microbial DNA was extracted from soil samples (or from litter-soil mixtures, see below) using the E.Z.N.A.® soil DNA Kit (Omega Bio-tek, Norcross, GA, U.S.) according to manufacturer’s protocols. The bacteria 16S and fungi 18S ribosomal RNA gene were amplified by PCR using primers 515F 5′-GTGCCAGCMGCCGCGG-3′, 907R 5′-CCGTCAATTCMTTTRAGTTT-3′and SSU0817F 5′-TTAGCATGGAATAATRRAATAGGA-3′ and 1196R 5′-TCTGGACCTGGTGAGTTTCC-3′ respectively, where barcode is an eight-base sequence unique to each sample. PCR reactions were performed in triplicate 20 μL mixture containing 4 μL of 5 × FastPfu Buffer, 2 μL of 2.5 mM dNTPs, 0.8 μL of each primer (5 μM), 0.4 μL of FastPfu Polymerase, and 10 ng of template DNA. Amplicons were extracted from 2% agarose gels and purified using the AxyPrep DNA Gel Extraction Kit (Axygen Biosciences, Union City, CA, U.S.) according to the manufacturer’s instructions and quantified using QuantiFluor™ -ST (Promega, U.S.). We calculated the Shannon diversity indices to represent the diversity of soil bacteria and fungi.

In addition, the CO_2_ and CH_4_ fluxes of soil was also measured. Concentrations of CO_2_ and CH_4_ were measured using a gas chromatograph (Agilent 7890 A, Santa Clara, CA). At first, we collected soils samples (at the beginning of experiment) or mixtures (2 weeks, 4 weeks, 6 weeks, 8 weeks) (about 10 g dry weight) from the CW by shovel or buckets by self-zip plastic bag. And they were putted in the glass bottle (100 ml). Before gas sampling, we sealed the glass bottle with airtight butyl rubber stoppers. After 24 h of incubation, the headspace gas of the glass bottle were sampled using airtight syringes. All gas samples were measured within 24 h after sampling.

During incubation, litter-soil mixtures were sampled every two weeks and then brought to the lab for the measurement of CO_2_ and CH_4_ fluxes. For the last sampling, the properties and microbial communities of litter-soil mixtures were again measured using the methods mentioned above. The whole experiment last two months from August 31, 2017 to November 2, 2017. After incubation, all the plastic buckets were removed from the CW to avoid the continuous disturbance for the CW.

### Statistical analysis

All data were checked for assumptions of normality and homogeneity of variance before analysis. We firstly compared the differences of initial litter traits, such as TN, TC, TP, C/N, C/P, N/P, ^13^C, ^15^N, among four submerged plant species. Secondly, we conducted one way ANOVAs to examine the differences of soil microbes (relative abundance and Shannon diversity index), TC, TN, ^13^C, ^15^N, CO_2_ and CH_4_ between the initial soil (before incubation) and the control treatment after two-month incubation in the constructed wetland (Fig. 1, ANOVA 1) and examine the effects of plant species on soil microbes (relative abundance and diversity), TC, TN, ^13^C, ^15^N, CO_2_ and CH_4_ after two-month litter mixing (Fig. 1, ANOVA 2). Thirdly, we analyzed the relationship between initial litter traits (TN, TC, TP, C/N, C/P, N/P, ^13^C, ^15^N) and the change value of soil characters (microbial diversity, TC, TN, ^13^C, ^15^N, CO_2_ and CH_4_) before and after two-month incubation using regression analysis respectively. In the end, the effects of plant species on microbial respiration (CO_2_, CH_4_) during two-month litter mixing were analyzed using repeated measure ANOVA. Differences between means were tested with Fisher LSD tests; effects were considered significant at *P* < 0.05. All the ANOVA analyses were conducted in SPSS Statistics (SPSS, Chicago, IL, USA), and regression analyses were conducted in R software 3.5.2 (R core Team)^54^.

## Supplementary information

Supporting information
