# Supplementary material for: The soil bacterial and fungal diversity were determined by the stoichiometric ratios of litter inputs: evidence from a constructed wetland
Source: Sci Rep. 2019 Sep 25;9:13813. doi: 10.1038/s41598-019-50161-9 (PMC6761198; doi:10.1038/s41598-019-50161-9)
Supplement: Supplementary file 1 — Supporting information [file 41598_2019_50161_MOESM1_ESM.pdf]

The soil bacterial and fungal diversity were determined by the stoichiometric ratios of litter inputs: evidence from a constructed wetland

Yunmei Ping[1,2,3]†, Xu Pan[1,2,3]†, Wei Li[1,2,3], Jinzhi Wang [1,2,3], Lijuan Cui[1,2,3]*

[1] Institute of Wetland Research, Chinese Academy of Forestry, Beijing 100091, China.

[2] Beijing Key Laboratory of Wetland Services and Restoration, Beijing 100091, China.

[3] Beijing Hanshiqiao National Wetland Ecosystem Research Station, Beijing 101399, China.

***** Correspondence: wetlands108@126.com or lkyclj@126.com; Tel.: +86-10-6282-4151

† These authors contributed equally to this work.

Appendix

Fig.S1 The changes of the microbial respiration (CO_2 and CH_4) of control treatment and soil with different litter species during experiment time (0 week, 2 weeks, 4 weeks, 6 weeks, and 8 weeks). Values are means ± S.E. (n = 3).

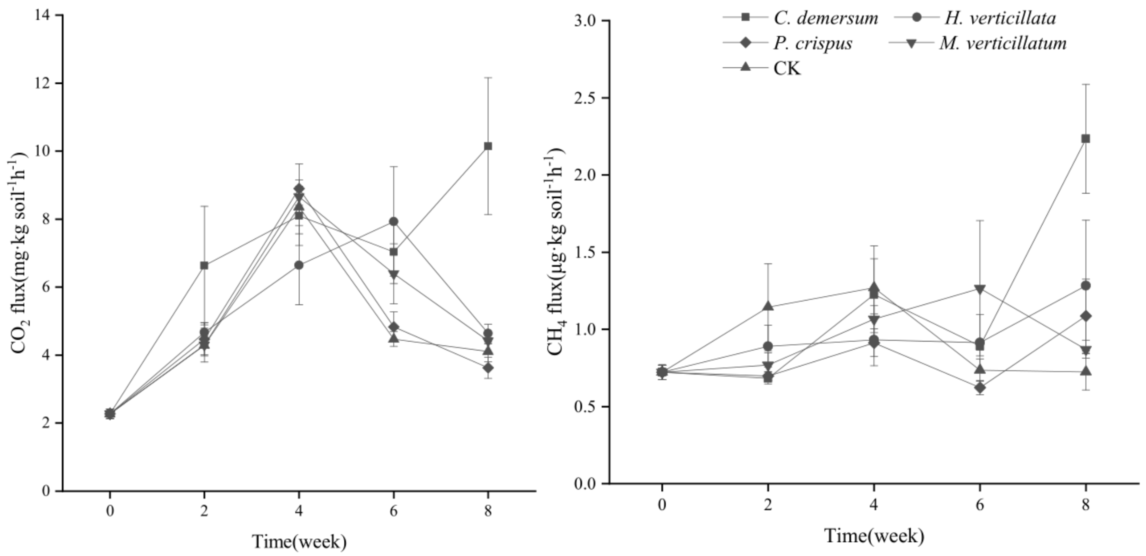

Table S1 One-Way ANOVA results for initial soil (without litters) microbial diversity, TC, TN, ^{13}C, ^{15}N, CO_2, CH_4 and the control treatment (CK) after two-month incubation in the constructed wetland.

	df	*F*	*P*
Shannon-bacteria	1	*4.90*	*0.07*
Shannon-fungi	1	0.01	0.94
TN	1	*4.35*	*0.03*
TC	1	0.63	0.46
^{13}C	1	1.68	0.24
^{15}N	1	0.66	0.45
CO_2	1	**33.14**	**0.00**
CH_4	1	0.01	0.98

Table S**2** Repeated measures ANOVA results for the effects of measurement time and litter species on fluxes of CO_2 and CH_4.

	df	**CO_2**		**CH_4**	
		F	*P*	*F*	*P*
Group 1:					
Measurement time(T)	5	**34.24**	**<0.01**	2.24	0.12
T*S	20	1.88	0.19	**3.18**	**0.01**
Group 2:					
Species(S)	4	*3.50*	*0.09*	1.37	0.35
